# Some Dangers Attending the Use of the Rectangular Position in Injuries of the Elbow-Joint

**Published:** 1893-11-04

**Authors:** A. Hanbury Frere


### SOME DANGERS ATTENDING THE USE OF THE RECTANGULAR POSITION IN INJURIES OF THE ELBOW-JOINT.

By A. Hanbury Frere, M.B.

The elbow is perhaps more interesting, and has been the subject of more discussion than any other joint in the body. Nor is this to be wondered at when we remember its importance, its complexity, and its mechanism, combining, as it does, the greatest elegance of form with the maximum of usefulness.

We have in the elbow the best example of an "osseously strong" joint, which, in the child, is specially liable to fracture. Injuries of the elbow form, therefore, an important group, and the difficulty of diagnosis, and the trouble attending the treatment of these cases, have long been a source of anxiety to the practitioner.

The practice of putting the arm up at a right angle in these cases is of very ancient date, and the points originally advanced in its favour are no less telling in our own day. Formerly employed in all injuries of the elbow, it is now usual to make an exception in fracture of the olecranon process of the ulna.

But a reference to the literature of the subject will show that this method, recommended by all text-books, is productive of results which leave much to be desired; in fact, the ordinary treatment of elbow fracture is very frequently followed not only by more or less diminution in the range of function, but also by deformity of the elbow-joint.

I have thought it might be well, therefore, to draw attention to certain dangers attending the use of the rectangular position, and in doing so one must keep constantly in view the natural movements and structure of the elbow-joint, and by associating certain facts regarding the normal elbow, it will not be difficult to see why this method has proved so unsatisfactory.

The elbow is a hinge joint, which is capable of flexion and extension only. It is in some respects comparable to the knee-joint, but whereas the knee is practically a straight hinge, the elbow is an oblique one, and herein lies one of the chief difficulties in the management of this joint. I need not here dwell upon the natural angularity of the upper limb, which has been the subject of special study by Pilcher, further than to say that the essential feature of the deformity seen after elbow fracture is an alteration in the normal outward obtuse angle at the elbow.

One of the great drawbacks to the rectangular position in cases of elbow fracture is the fact that, with the arm at a right angle, we cannot be sure that we are keeping up accurate coäptation of the fragments. As Lauenstein* points out, marked deformity of the elbow is entirely concealed by flexing the limb, and however much the fragments may be displaced we shall never be aware of the fact so long as the arm is bent.

Again, with the arm at a right angle, the parts in

* Verhandlungen der Deutschen Gesellschaft fur Chirurgie. April, 1888.

front of the joint are relaxed. Thus by contraction important structures may become entangled in the callus, so that, when the arm is straightened, the vessels and nerves are in danger of being torn.

So long as the arm is straight the muscles around can only approximate the articular surfaces; so soon, however, as we flex the forearm, however slightly, the anterior group of muscles come into play, and may aid in causing displacement.

It is, moreover, almost impossible to fix the arm with any degree of certainty in the bent position, for the weight of the forearm alone is sufficient to loosen any of the ordinary forms of dressing, and then, as Hamilton* has shown, there occurs a movement of the lower fragment upon the upper, which must interfere with the healing process.

If a normal arm be put up at a right angle it very soon becomes stiff; a temporary false anchylosis occurs, which in an injured elbow is likely to be more marked. Such stiffness will be most inconvenient, since, when we begin passive motion, movement may take place at the seat of fracture instead of at the elbow-joint.

Cases have occurred in which, when the arm was bent to a right angle, flexion took place entirely at the seat of fracture, and the mistake was not found out till some time after, when it was found that the elbow-joint was still extended though apparently flexed.

When the limb has been dressed at a right angle we are very apt to have trouble in performing passive motion, because when we straighten the arm, we do so in opposition to the powerful anterior group of muscles, and if the soft parts have contracted at all there is the danger of lighting up some inflammatory action, which may cause much pain to the patient.

It must be remembered that in elbow fracture the fragments are almost entirely at the mercy of the bones of the forearm. In this connection Allis† has demonstrated very clearly how the ordinary methods of fixation may, by their pressure on the bones of the forearm, which are not on the same level, influence very materially the position that the fragments will take up. Hence by elevation of the internal condyle, or by depression of the external, or by both, as in separation of the lower humeral epiphysis, the direction of the axis of the lower articular surface of the humerus is altered, thus bringing about deformity.

In backward displacement of the fragments the bent position would seem to be indicated, but it must be remembered that when we flex the arm we may carry the lower fragment too far forward, and, as we have seen, such an accident would not be detected so long as the limb is bent.

Then so long as the routine treatment of these cases is to put the arm up at a right angle, there is a danger of fracture of the olecranon, which may be overlooked, being dressed in the rectangular position with disastrous results.

But there is another danger attending the ordinary use of the rectangular position, which, so far as I can discover, was first pointed out by myself in the *Provincial Medical Journal*.‡ As a result, says Morris § of the oblique plane of motion of the ulna on the humerus, the hand in flexion is carried towards the middle third of the clavicle, and any further inclination of the hand towards the median line of the body is caused, not by anything in the construction of the elbow, but by rotation of the humerus at the shoulder. If then we wish to bend the arm to a right angle across the front of the body, we do so by rotation of the humerus at the shoulder. Here then is a danger attending the use of the rectangular position in cases of elbow fracture, for the rotation necessary to bring the arm into

* On Fractures and Dislocations.

† Annals of Anatomical and Surgical Society. Brooklyn. Vol. II. No. 8. 1880.

‡ Jan. Mar. and April. 1892.

§ Anatomy of the Joints of man.

this position is likely to occur, not at the shoulder, but at the seat of fracture, thus causing displacement.

It is interesting to note in this connection that, as Cathcart * has shown, the ulna participates in pronation and supination of the forearm, but this movement of the ulna is also due to rotation of the humerus at the shoulder.

But, as I pointed out in the *Lancet*,† there is a great difference between flexing to a right angle a supinated arm on the one hand, and a pronated arm on the other, for this reason, viz., when the supinated limb is bent to a right angle the rotation of the humerus occurs during the act of flexion; when, however, the pronated limb is bent to a right angle there is no rotation of the humerus, because rotation has taken place during the act of pronation, and while the arm is still extended, an important point in the case of elbow fracture, since with the limb straight we are able to see that no displacement shall take place from the necessary rotation of the humerus. The pronated limb is therefore quite ready to be bent to a right angle without any movement other than flexion, and if we desire to dress the arm at a right angle we must be careful, in setting the fracture, to keep the forearm prone, in order to avoid the risk of displacement.

I am the more inclined to attach importance to this point from the fact that it would seem to give us an explanation of what Hippocrates meant, when he taught that it was bad to bind the arm in the extended and supine, or the extended and semi-prone (Archer's) position, because, when the arm thus bandaged is bent to a right angle, the bones at the elbow will assume a different attitude to that in which they were bound up; but if the arm is bandaged in the extended and prone position, when we bend to a right angle the bones at the elbow will be in the same attitude as that in which they were bound up.

If I am right in my interpretation of Hippocrates' words‡, I hold that the rectangular position, as employed in the present day, is not in accordance with the teaching of those from whom we may be supposed to have learned of its use, and this may be one great reason why the rectangular position has proved so unsatisfactory in our hands. Be this as it may, we find at the present time the most opposite opinions expressed as to the treatment, not only of the initial lesion, but also during the process of repair. Thus the question, "How soon should the joint be moved?" has given rise to much discussion, and this leads me to say that the so-called passive motion, as so often employed, is calculated to do great harm, inasmuch as it is frequently practised without any regard to the natural movements of the elbow-joint. I hold that there must be no wrenching or forcing of the limb in a direction contrary to nature, and, when the joint is moved for the first time, the hand should undoubtedly be carried towards the middle third of the clavicle in flexion, and downwards and outwards from that point in extension, in order that the joint may travel along its natural path.

It is to be feared that our progress in the treatment of these cases has been greatly impeded by a too rigid devotion to authority and established routine, which as Paris§ long ago pointed out, has always been the means of opposing the advancement of natural truths, and it has perpetuated many stupendous errors. It is difficult to see what else can have led surgeons to persist in the employment of a method, which, as we have seen, is most favourable to the very danger they most dread.

To recapitulate shortly, in dressing the arm at a right angle the chief danger is displacement, which may

* Journ. Anat. and Phys. Vol. XIX. 1885.

† October 1st, 1892.

‡ Adams' Translation.—Sydenham Soc.

§ Pharmacologia. 8th Ed. 1833.

take place in various directions, viz.: (1) Upwards or downwards in fracture of the condyles; (2) side to side rocking in separation of the epiphysis; (3) antero-posterior; (4) rotatory unless the arm be kept prone before bending to a right angle. Whilst all of these will cause deformity, none of them will be detected so long as the arm is bent. The rectangular position is an unnatural one, and, in my opinion, constitutes a most dangerous routine practice.

In conclusion let me urge a more careful consideration of the natural movements and structure of the elbow-joint, not only in setting the fracture, but also while performing passive motion, during the process of repair.

